# Self-Powered Edible Defrosting Sensor

**DOI:** 10.1021/acssensors.2c01280

**Published:** 2022-10-12

**Authors:** Ivan K. Ilic, Leonardo Lamanna, Daniele Cortecchia, Pietro Cataldi, Alessandro Luzio, Mario Caironi

**Affiliations:** Center for Nano Science and Technology@PoliMi, Istituto Italiano di Tecnologia, via Giovanni Pascoli 70/3, Milano 20133, Italy

**Keywords:** sensors, detectors, edible electronics, galvanic cells, ionotronics, defrosting, ionochromism, food

## Abstract

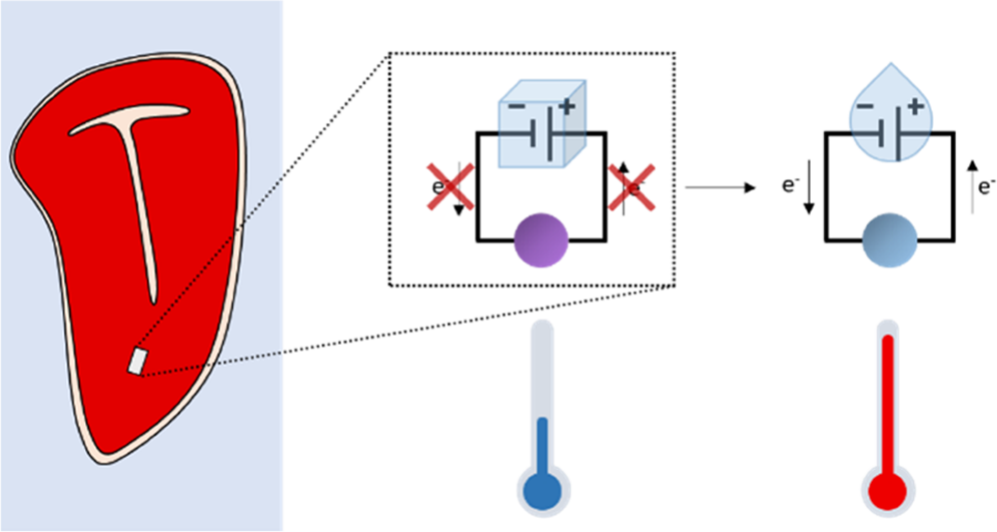

Improper freezing of food causes food waste and negatively impacts the environment. In this work, we propose a device that can detect defrosting events by coupling a temperature-activated galvanic cell with an ionochromic cell, which is activated by the release of ions during current flow. Both the components of the sensor are fabricated through simple and low-energy-consuming procedures from edible materials. The galvanic cell operates with an aqueous electrolyte solution, producing current only at temperatures above the freezing point of the solution. The ionochromic cell exploits the current generated during the defrosting to release tin ions, which form complexes with natural dyes, causing the color change. Therefore, this sensor provides information about defrosting events. The temperature at which the sensor reacts can be tuned between 0 and −50 °C. The device can thus be flexibly used in the supply chain: as a sensor, it can measure the length of exposure to above-the-threshold temperatures, while as a detector, it can provide a signal that there was exposure to above-the-threshold temperatures. Such a device can ensure that frozen food is handled correctly and is safe for consumption. As a sensor, it could be used by the workers in the supply chain, while as a detector, it could be useful for end consumers, ensuring that the food was properly frozen during the whole supply chain.

## 1 Introduction

Modern society produces plenty of food that frequently goes to waste. An estimated 88 million tons of food waste is produced every year in the European Union alone,^1^ creating a substantial environmental impact, the equivalent of 186 million tons of carbon dioxide that is slightly higher than the annual CO_2_ emission of countries such as the Netherlands, Venezuela, or Pakistan.^2^ The impact of animal-derived foods is exceptionally high. Indeed, the production of such foods requires massive amounts of land, food, energy, and water.^3^ Freezing food, especially meat, is highly effective in increasing its shelf life, and thus, it massively reduces waste, preserving food quality and safety.^4−7^ However, food quality is affected during freezing and thawing,^7^ and this is especially dangerous during repeated freezing and thawing cycles of meat as it can significantly increase the count of many pathogenic microorganisms.^8^ Therefore, it is crucial to deliver frozen meat to the end consumer without thawing. Moreover, preservation at subzero temperatures is exploited to extend the shelf life of other types of food, such as vegetables, fruits, and sweets. Many medical preparations are stored at low temperatures as well, such as mRNA-based vaccines, which have to be stored at low temperatures (i.e., −60 °C) to be correctly preserved.

Commonly used temperature sensors in the food supply chain measure the temperature of food containers (e.g., refrigerator). These sensors are designed to monitor food conditions during the supply chain but cannot be used directly by the end consumers. Furthermore, the food quality is modeled based on the sensor reading.^9^ These facts sparked interest in developing time–temperature indicators,^10−13^ simple devices that monitor for how long the food item has been exposed to a temperature above the set threshold, producing a feedback signal. Monitoring the time of exposure to excessive temperatures is essential as food items or medical preparations usually withstand elevated temperatures for short periods without undergoing any change. The signal produced by these devices is predominantly based on a color change for immediate visual recognition. Previously exploited phenomena for time–temperature indicators include dewetting thin films at various temperatures,^10^ chronochromic heteroepitaxy of plasmonic nanocrystals,^11^ bathochromic shifts of polymer absorption due to supramolecular rearrangement,^12^ and pigment degradation.^13^ However, all of these devices exploit phenomena that occur above the melting point of water and therefore are not suitable to monitor defrosting events. To our knowledge, there is a lack of simple, low-cost sensors, which can provide information about defrosting events to the retailer and end consumers. In addition, the sensors used for food monitoring have to be made out of nontoxic materials to ensure that they can be safely in contact with food. Furthermore, they need to be biodegradable in case of mass production.

The new paradigm of edible electronics, emerging in the more general framework of green electronics,^14,15^ exploits the inherent electronic properties of food and food additives.^16,17^ This makes it optimal for electronic devices in close contact with food as it aims to develop devices safe for human consumption. There is a great interest in such devices in medicine, as they could in part replace or complement current ingestible electronics technologies, and also for food monitoring. To date, there have been a few attempts toward edible devices, such as batteries,^18^ supercapacitors,^19,20^ fuel cells,^21^ microphones, radio frequency filters,^22^ and sensors.^22,23^ Furthermore, an edible conductive paste was recently developed, which could replace metallic wiring.^24^ An edible electronic system would allow for safe food monitoring, giving in situ information about the state of the food at any stage of the supply chain or to the end consumer. The development of food-compliant devices will give information inaccessible to the available sensors, not attached directly to food but placed in the vicinity of the food.

To date, no temperature sensors made from food-grade materials and safe to be eaten have been reported.

Here, we propose the first fully edible temperature sensor. It is based on a galvanic and an ionochromic cell. Previously, galvanic cells were exploited as detectors in ingestible electronics: a galvanic cell, assembled without an electrolyte, would start generating electricity only after being immersed in gastric acid—an electrolyte. Therefore, such a cell was acting as the detector for ingestion.^25^ Similarly, galvanic cells have been exploited to form thermal batteries, which start generating electricity only at a specific temperature.^26−28^ Thermal batteries have electrolyte cells filled with solid salts. Solids have significantly lower ionic conductivity than liquids, as the molecules organize in a state of low mobility, and therefore, a galvanic cell filled with a solid salt does not generate current. However, the salt conducts ions upon melting, and the galvanic cell generates a current. Therefore, galvanic cells filled with pure salts act as sensors whose activation temperature equals the melting point of the salt, rarely under 100 °C, in the electrolyte chamber. Inspired by a previously reported biodegradable battery that used a magnesium anode and a nonedible transition metal as the cathode,^29^ we propose an edible galvanic cell, using a magnesium anode and a gold cathode in an aqueous electrolyte. It provides no current while the electrolyte is frozen, supplying the current only when the electrolyte is defrosted. The temperature of activation of the current flow can be modulated by changing the freezing-point depression employing salts with different solubilities. Such a galvanic cell is used as a power source for an ionochromic cell, creating the first edible defrosting sensor. The ionochromic cell, which is an electrochemical cell containing ionochromic compounds in the electrolyte, cannot undergo electrochemical reaction spontaneously due to the mismatch of the reduction potentials of the electrodes. When the current is applied to such a cell, an irreversible color change takes place. Thus, the edible defrosting sensor, containing the galvanic cell and the sensor, can detect defrosting events through simple color changes. Furthermore, such a color change is quantifiable, allowing us to determine the length of exposure to the defrosting temperatures. Implementing such a sensor in the cold supply chain can ensure that the frozen products are properly handled from the first frosting till the end consumer. Therefore, this device can also be seen as a novel time–temperature indicator, which exploits defrosting phenomena as a trigger, working at significantly lower temperatures than previously reported devices.

## 2 Results and Discussion

### 2.1 Development of a Temperature-Activated Edible Galvanic Cell

**Figure 1:**
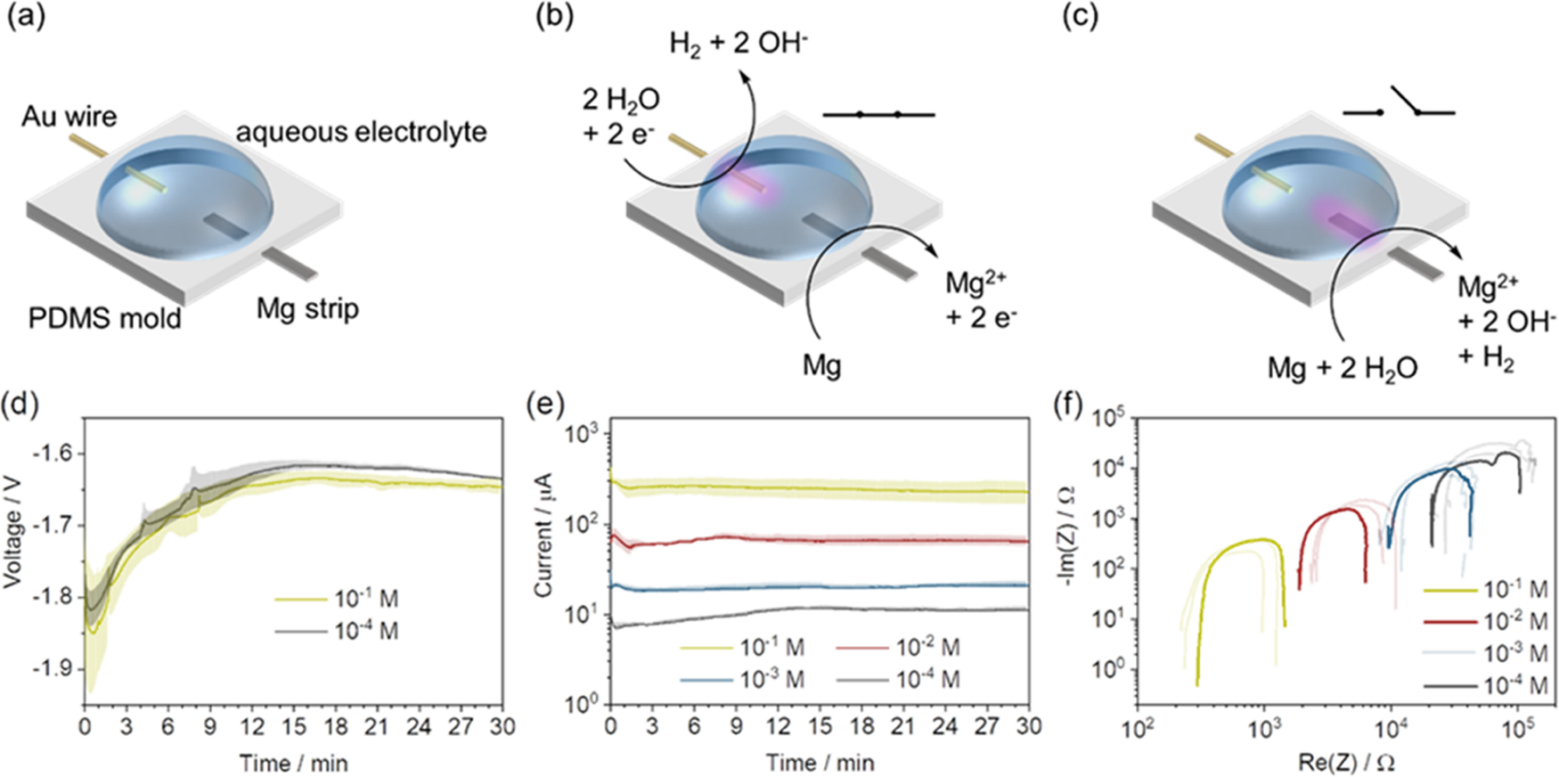
Mg–Au galvanic cell with NaCl as an electrolyte. (a) Electrochemical galvanic cell used for the investigations with Au wire and Mg strip as the electrodes, polydimethylsiloxane (PDMS) chamber as an auxiliary scaffold, and an aqueous solution of NaCl as the electrolyte. (b) Electrochemical reactions occurring in the galvanic cell operating in a closed circuit. Purple shade indicates a local high concentration of OH^–^. (c) Chemical reactions in the galvanic cell in an open circuit. (d) Open-circuit voltage in the galvanic cells filled with the 10^–1^ and 10^–4^ M aqueous NaCl solutions. (e) Chronoamperometry of the closed-circuit galvanic cells filled with different aqueous NaCl solutions (ranging from 10^–1^ to 10^–4^ M). (f) Nyquist plot of the galvanic cells filled with the solutions in panel (e) measured from 1 mHz to 1 MHz.

A galvanic cell, which can undergo spontaneous discharge, was developed by adapting edible active materials. With the only aim of facilitating the experiments, a PDMS chamber, pierced with the electrodes and filled with the electrolyte, was adopted (Figure 1a). An aqueous solution of NaCl was used as the electrolyte. A Mg strip was used as the negative electrode due to its low standard reduction potential of −2.4 V vs standard hydrogen electrode (SHE). A Au wire was used as the electrocatalyst for water reduction: the reduction occurring at the cathode has a potential of 0 V (pH 0) or −0.4 V (pH 7) vs SHE according to the Nernst equation. Therefore, since we are working in a NaCl solution at pH 7, the theoretical voltage expected from such a cell is 2.0 V. The reactions in the cell can be summarized as
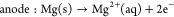

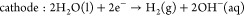


When the Mg and Au electrodes are connected, closing the circuit, the electrochemical reactions occur (Figure 1b). During the cell operation, Mg gets oxidized, releasing Mg^2+^, and two electrons per ion, while the water gets reduced, accepting one electron per water molecule and forming gaseous H_2_. We investigated the reactions occurring in the galvanic cell by filling the cell with the electrolyte containing phenolphthalein. This common pH indicator changes its color from colorless to purple in a basic environment. The experiment produced evidence of OH^–^ formation close to the Au electrode (Figure 1b, the experiment is discussed in the supporting information (SI), Figure S1, Video S1).^30^

Nevertheless, the low reduction potential of Mg can result in a thermodynamic reaction with water in open-circuit conditions, forming Mg^2+^ and H_2_ (Figure 1c). The reaction of Mg with water indeed occurs in the open circuit, with purple color developing around the Mg electrode in this case. However, it is significantly less pronounced during the closed-circuit cell operation, as shown in Figure S1.

The galvanic cells were investigated using NaCl electrolyte with different concentrations between 10^–1^ and 10^–4^ M. The open-circuit voltage is independent of the electrolyte concentration (Figures 1d and S2), reaching a value of around −1.65 V. The discrepancy between the theoretical (2 V) and the experimental value can be explained by the large overpotential of the water reduction on the Au surface.^31^ The closed-circuit current delivered by the galvanic cell depends on the salt concentration in the electrolyte, as a higher ionic conductivity is achieved with increasing concentration (Figure 1e). This trend is confirmed by the Nyquist plots (Figure 1f), measured from 1 mHz to 1 MHz by electrical impedance spectroscopy. The higher concentration of the electrolyte decreases both the solution resistivity and the charge transfer device resistance, leading to higher current flows at a constant voltage.

**Figure 2:**
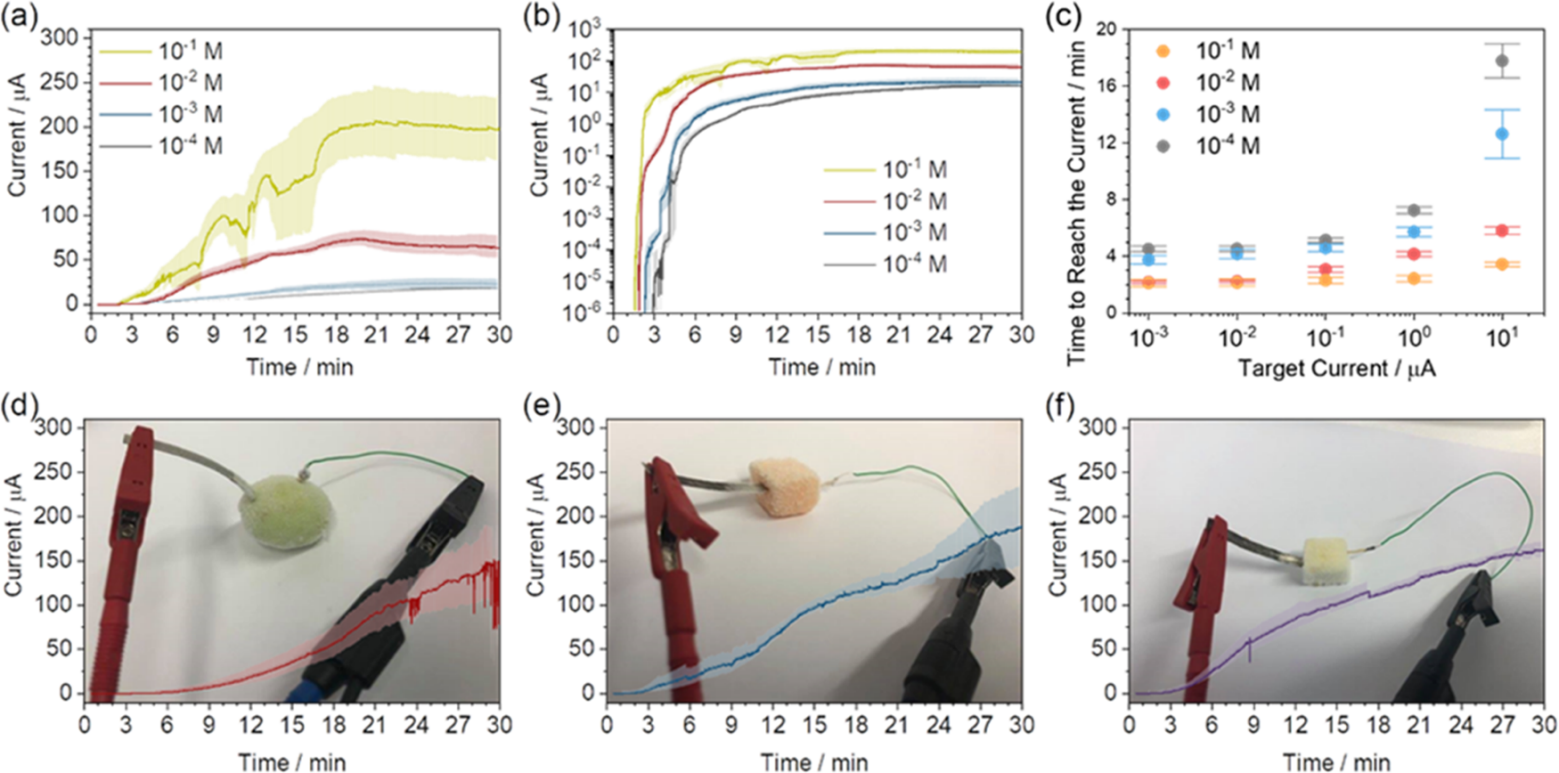
Defrosting of edible galvanic cells with different NaCl electrolyte concentrations. (a, b) Chronoamperometry of the galvanic cells filled with different aqueous NaCl solutions during defrosting in (a) logarithmic scale and (b) linear scale. (c) Time needed by the galvanic cells to reach the target current (10^–3^, 10^–2^, 10^–1^, 1, and 10 μA). (d–f) Defrosting of galvanic cells with fruits as the electrolytes: grape, melon, and apple, respectively.

Next, we tested the temperature activation of such galvanic cells. Galvanic cells with variable NaCl electrolyte concentrations were placed in a freezer at −80 °C, immediately after the addition of the electrolyte. After the temperature was reduced to −80 °C, the galvanic cells were taken from the freezer to room temperature, and the current was measured using the chronoamperometry technique (Figure 2a). It took about 30 s to start the measurement from the time the cells were taken out of the freezer. Therefore, the first 30 s are not shown for any measurement. In the beginning, irrespective of the electrolyte, all of the galvanic cells showed no detectable current flow (the potentiostat can reliably detect current as low as 10^–4^ μA). After a few minutes, the current started rising abruptly in all cells, due to electrolyte melting. The cell activation time (Figure 2a), i.e., the time at which an abrupt current increase takes place, can be tuned by changing the electrolyte concentration, ranging from 1.5 to 3 min from the highest (10^–1^ M) to the lowest (10^–4^ M) concentration employed. This allows modification of the response time of the galvanic cell, as it can be set to tolerate up to a few minutes without activation. After activation, the rate of current increase and the maximum reached current also depend on the electrolyte concentration. This is expected as cells with higher electrolyte concentrations deliver higher currents. In Figure 2b, it is visible how long galvanic cells take to reach arbitrary current thresholds of 10^–3^, 10^–2^, 10^–1^, 1, and 10 μA. The lower the concentration of the electrolyte, the longer it takes for the galvanic cell to reach the target current. Therefore, the response time of the sensor can also be tuned with a simple change of the concentration of the electrolyte. As reported in Figure 2b, except for the two lowest concentrations and 10 μA threshold, the standard error in these measurements is rather small (less than 20 s).

Liquid electrolytes, such as an aqueous solution of NaCl, are good ion conductors, but they have to be contained in scaffolds. However, many foods, such as fruits, are natural electrolyte-rich scaffolds. Their fibrous structure allows them to easily accommodate metals, while their juices are filled with ions, making them suitable electrolytes. Following the same rationale that guided us in devising aqueous-based galvanic cells as the base for defrosting sensors, we utilized the natural juices of fruits as electrolytes. Here, we demonstrate that grape (Figure 2d), melon (Figure 2), and apple (Figure 2f) can be used not only as electrolytes but also as scaffolds for edible galvanic cells. The devices were simply prepared by piercing a piece of fruit with metallic electrodes described in Figure 1. All of the fruits showed defrosting behavior similar to a 0.1 M aqueous solution of NaCl. They all yielded similar currents, indicating the relatively comparable electrolyte nature of different fruits. Therefore, we propose that pieces of low-cost fruits could be used as natural scaffolds and electrolyte reservoirs, directly attached to high-end foods, such as meat and fish, to monitor their correct storage. However, fruit-based cells could pose a difficulty in tunability, as the electrolyte concentrations cannot be easily changed, limiting them to niche applications. Therefore, in the following, we will adopt PDMS chambers, and lastly, we will propose an edible alternative.

**Figure 3:**
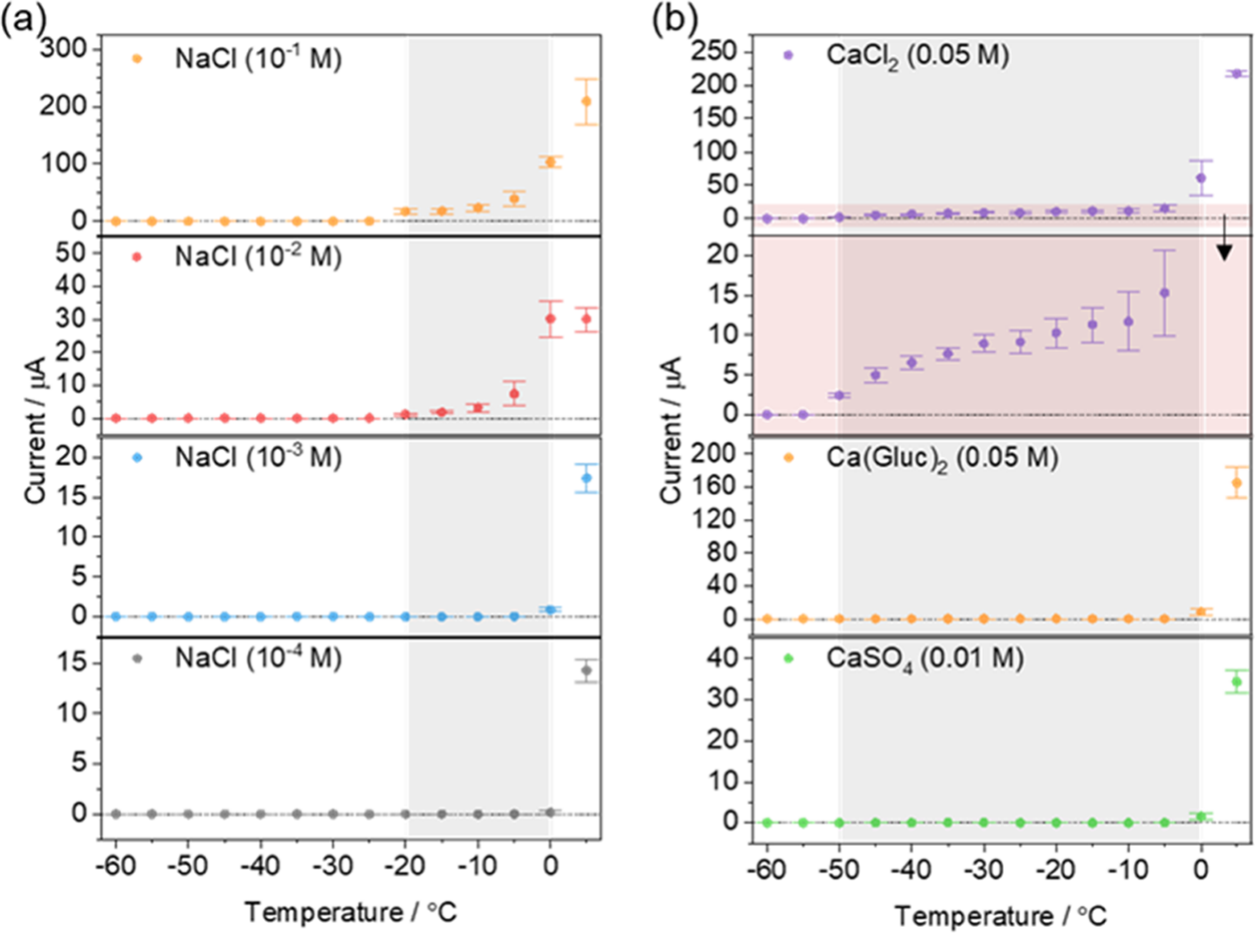
Currents produced by the galvanic cells containing (a) NaCl electrolyte and (b) Ca-based electrolyte at different temperatures during heating of the cooled system.

To better understand how the current develops during defrosting, its change was recorded during the melting of the electrolyte while monitoring the temperature, adopting modified galvanic cells, assembled as shown in Figure S3 and as described in the supporting information. The change in the temperature was buffered by immersing the galvanic cells in ethanol, cooling the device in the freezer, and the temperature of the ethanol was monitored. All of the galvanic cells showed no current at temperatures below −20 °C (Figure 3a). Two devices with the lowest concentration of NaCl, 10^–3^ and 10^–4^ M, exhibited no current until the melting point of water, 0 °C. However, the devices with the higher concentrations, 10^–1^ and 10^–2^ M, exhibit current flow already at a sub-melting point temperature of −20 °C. Freezing-point depression in the solutions is a well-known phenomenon, proportional to the cryoscopic constant and the molality of the ions in the solution. The melting/freezing of NaCl solutions occurs at a temperature of −20 °C just in a saturated condition (6.15 mol kg^–1^).^32^ For lower concentrations, water phase transition (freezing) should be negligible, smaller than 0.4 °C for 10^–1^ M NaCl. A similar effect was observed before, but the authors did not comment on it.^33^ Therefore, freezing-point depression alone cannot explain our observations.

To understand the role of freezing-point depression in the current onset at low temperatures, we compared salts with different solubilities. As NaCl is very well soluble in water (6.15 mol kg^–1^ at 25 °C^32^), we compared it with salts of lower solubility, namely, calcium gluconate (Ca(gluc)_2_, 0.0654 mol kg^–1^ at 25 °C^34^) and calcium sulfate (CaSO_4_, 0.015 mol kg^–1^ at 25 °C^32^). Furthermore, we compared it with CaCl_2_, a salt with solubility even higher than NaCl (7.32 mol kg^–1^ at 25 °C^32^). As CaCl_2_ dissociates to three ions when dissolved, as compared to two ions for NaCl, its freezing-point depression is even greater, reaching values below −50 °C. Concentrations of Ca(gluc)_2_ and CaSO_4_ were chosen to be near the saturation level, at 0.05 and 0.01 M, respectively, while 0.05 M CaCl_2_ was used as NaCl showed percolation behavior already at similar levels. At room temperature, cells based on Ca(gluc)_2_ and CaSO_4_ solutions exhibit similar currents, around 80 μA, while CaCl_2_ solution shows a much higher current of over 200 μA (Figure S4). During slow defrosting, CaCl_2_ solution-based cell exhibits no current at temperatures under −50 °C, but at higher temperatures, the current starts rising slowly, with an abrupt increase at 0 °C (Figure 3b). Furthermore, neither Ca(gluc)_2_ nor CaSO_4_ solution exhibited any conductivity at subzero temperatures. This behavior indicates that, besides the change in salt concentration, the use of different salts offers an additional knob to tune the activation temperature of the galvanic cell.

**Figure 4:**
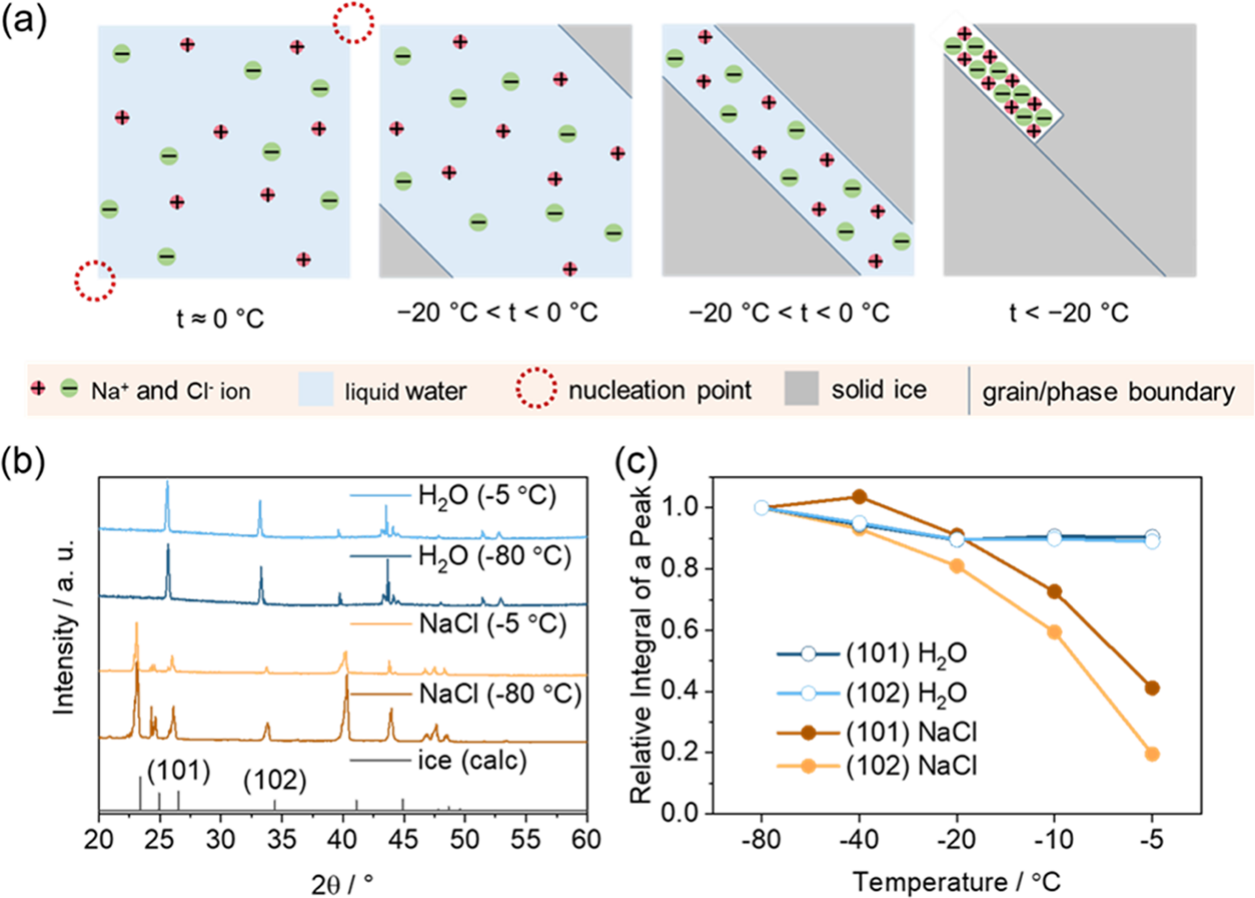
(a) Freezing of nonsaturated NaCl solution. Liquid water contains Na^+^ and Cl^–^ ions, and at 0 °C, nucleation points form. Under 0 °C, solid ice grows from the nucleation points, causing an increase in the ion concentration in the remaining liquid water, until the NaCl solution saturates. Solid ice continues to grow, expelling the remaining Na^+^ and Cl^–^ ions in the form of NaCl crystals. (b) X-ray diffractograms of water and 0.1 M aqueous solution NaCl at −80 and −5 °C and diffractogram of ice, as calculated.^36,51^ (c) Crystallinity of water and NaCl solution at different temperatures, calculated as integrals of the reflections.

Here, we propose a mechanism to explain the observed current change at the temperature at which the current abruptly rises. For clarity, we will first explain the events occurring during the freezing of the electrolyte solution, assuming that the opposite set of events occur during defrosting. The proposed mechanism is based on the current observed already at −50 °C for solutions of CaCl_2_ and −20 °C for NaCl; however, as an example, we use the solution of NaCl. An aqueous solution of NaCl contains hydrated Na^+^ and Cl^–^ ions. At temperatures below 0 °C, ice crystals start growing from nucleation points. The newly formed ice expels Na^+^ and Cl^–^ to the solution, ultimately creating saturated regions of NaCl that have a freezing point of −20 °C (Figure 4a). The opposite process occurs during melting, with pools of saturated NaCl solution forming already at −20 °C. When the NaCl is concentrated enough (10^–1^ and 10^–2^ M), the saturated pools percolate, forming a pathway for ions, causing the current flow in the galvanic cell already at −20 °C. At lower concentrations (10^–3^ and 10^–4^ M), there is no percolation network at subzero temperatures, and therefore, the current develops only after the system has been exposed to temperatures above 0 °C.^35^

The proposed mechanism was proved by performing X-ray diffraction (XRD) measurements, comparing pure water and 0.1 M aqueous solution of NaCl at different temperatures (Figure 4b). The sample was frozen at −80 °C and subsequently heated up to −40, −20, −10, −5, 0, and 10 °C (Figure S5). The amorphous-like diffraction pattern exhibited by the samples at 0 and 10 °C indicates water in the liquid phase. At temperatures below 0 °C, the samples show sharp reflections, indicating a crystalline nature. The reflections can be ascribed to the crystal structure of ice,^36^ with a slight shift toward wider angles, which increases at lower temperatures due to lattice contraction. The relative intensity of the different reflections changes once the salt is added to the water, an effect that can be ascribed to the modification in the growth habit planes and to morphological evolution of the ice crystals.^37−39^ However, both water and 0.1 M aqueous solution of NaCl exhibit (101) and (102) reflections at 27 and 34° angles, respectively. While the intensity of reflections of water remains nearly constant for temperatures up to 0 °C, this is not the case for the ice in 0.1 M NaCl aqueous solution, where the intensity of the diffraction peaks starts to decline for temperatures above −40 °C. Considering that the integration of the diffraction peaks is proportional to the crystallinity of the sample (Figure 4c), we conclude that the crystallinity of 0.1 M aqueous solution of NaCl decreases rapidly above −20 °C, as a result of the partial melting at subzero temperatures caused by the presence of NaCl-rich domains at the intergrain regions. To our knowledge, this is the first time the electrochemical conductivity of ice was correlated with the crystallinity of ice, an important insight into the mechanism by which the semifrozen aqueous solution conducts electricity. At −20 °C, the conductivity of the electrolyte increases significantly, as a layer of a liquid saturated solution of NaCl, formed between the grains, starts enabling ion percolation between the electrodes; thus, the liquid layer serves as the electrolyte, resulting in measurable current.

So far, we have shown that it is possible to tune the response temperature of edible galvanic cells by the accurate choice of the electrolyte salt and its concentration. In this way, we have demonstrated the tunability of the activation temperature to −50, −20, and 0 °C.

### 2.2 Edible Ionochromic Cell

**Figure 5:**
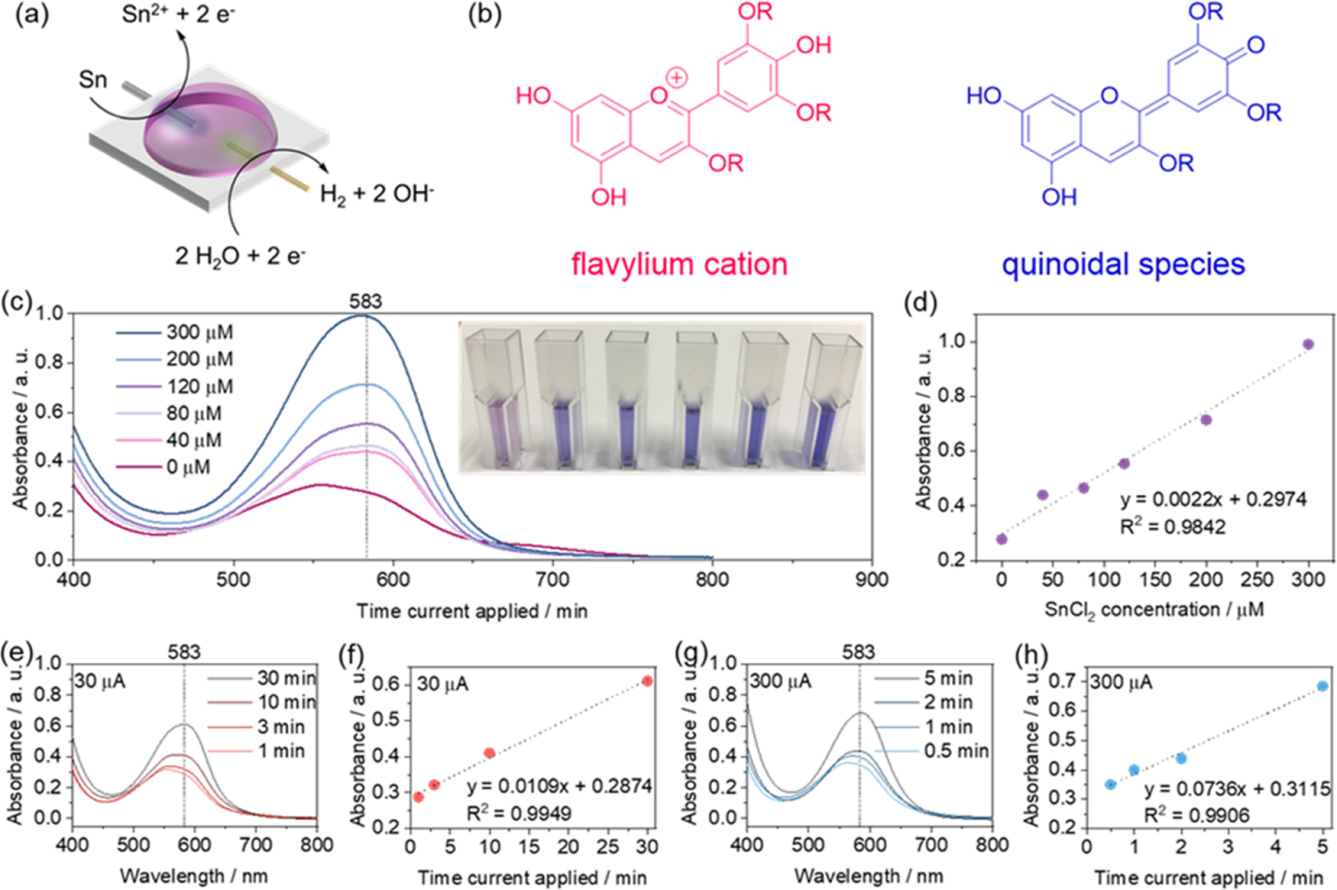
Edible ionochromic cell based on red cabbage juice. (a) The ionochromic cell consists of Au and Sn wires as electrodes. (b) Differently colored species of anthocyanins occur in different chemical environments.^43^ (c) Color shift of red cabbage juice upon the addition of a small portion of SnCl_2_ solution in ethanol as measured by ultraviolet–visible (UV–vis) spectroscopy (a photograph of the corresponding solutions in vials is presented in the inset). (d) Linear fit of the absorbance at 583 nm as a function of Sn^2+^ concentration. (e–h) Ionochromic cells assembled from 0.1 M aqueous solution of NaCl with Sn and Au wires as electrodes upon passing current. (e) UV–vis spectra of the electrolyte after passing 30 μA for different time periods. (f) Linear fit of the absorbance at 583 nm as a function of time the current was applied. (e) UV–vis spectra of the electrolyte after passing 300 μA for different time periods. (f) Linear fit of the absorbance at 583 nm as a function of time the current was applied.

As previously shown, the current of our edible galvanic cell can give direct information related to the exposure temperature (when above a defined limit) and consequently time. However, the detection of the current needs real-time readers (i.e., amperometers) and information storage strategies that are not simply implemented in the cold supply chain with edible electronics. Moreover, the information they provide could be challenging to access, especially for the end consumer. A feedback mechanism that does not need real-time monitoring and provides nonvolatile information on the “history” of the temperature of the monitored product would be ideal for an actual application of such technology in the food supply chain. Thus, linking the current produced by the galvanic cell to an irreversible color change, directly proportional to the developed current and the time, would be helpful. Furthermore, this sensor response could be quantified qualitatively (as a detector) with the color change directly by the end consumer. Alternatively, quantitative reading of the information stored in the sensor could be performed with a ultraviolet–visible (UV–vis) absorption setup during the supply chain. For this purpose, we developed an edible ionochromic cell, which is actually a galvanic cell with a mismatch in the electrode potentials and ionochromic species in the electrolyte. The cell was based on Sn and Au electrodes. Sn has a rather high standard reduction potential, as the Sn^2+^/Sn redox pair undergoes redox reaction at around −0.13 V vs SHE. Therefore, Sn cannot react with neutral water (reduction potential at around −0.4 V vs SHE) nor form galvanic cells, capable of spontaneous discharge, with water reduction as a cathodic reaction. However, after the current is supplied to the cell, the Sn^2+^ ions are released in its electrolyte (Figure 5a). As the electrolyte is formed from red cabbage juice, the ionochromic anthocyanins in the electrolyte change color from purple to blue upon interaction with Sn^2+^.

The deep purple color of red cabbage comes from water–soluble anthocyanins. Anthocyanins consist of a sugar and an anthocyanidin species.^40^ They are sensitive to the chemical environment, changing colors based on the chemical trigger, such as the pH of the solution (Figure S6).^41,42^ At low pH values, red-colored flavylium cations can be observed, while blue quinoidal species occur at slightly higher pH values (Figure 5b).^43^ However, the change of color can be accompanied not only by a pH change but also by the introduction of metal cations, such as Sn^2+^, which form complexes with anthocyanins.^43,44^ Blue complexes of Al^3+^ with anthocyanins were ascribed to the quinoidal form. Intriguingly, such complexes were found to exhibit higher stability to oxidation than the pristine anthocyanins, as the complexation protects the quinoidal form from oxidation.^45,46^ The addition of SnCl_2_ solution to red cabbage juice showed an increase in the adsorption at 583 nm (Figure 5c), proving the ionochromic properties. Absorbance at 583 nm shows a linear relationship with the amount of SnCl_2_ added (Figure 5d), as predicted by the Beer–Lambert law
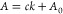


where *A* is the absorbance of the sample, *c* is the concentration of the absorbing species, *k* is the constant related to the cell, and *A*_0_ is the absorbance due to other species (as red cabbage juice contains a variety of organic molecules). Therefore, complexation of Sn^2+^ with anthocyanins from red cabbage can be used to detect Sn^2+^ in the solution and thus the current passing through the galvanic sensor provided by the galvanic cell, as described above.

Based on this mechanism, an ionochromic cell was built using Au and Sn wires as electrodes and 0.1 M NaCl solution in red cabbage juice as the electrolyte (Figure 5a). Glycerol was added to the electrolyte to reduce the freezing point,^47^ ensuring that the electrolyte in the galvanic sensor is not limiting the electron flow at low temperatures, i.e., at the operative temperature that we envision for our defrosting sensor. Upon applying 30 μA to such a system, the color of the electrolyte changes as indicated by the UV–vis spectra (Figure 5e). The enhancement of the absorption at 583 nm is linear with the time for which the 30 μA current is applied (Figure 5f), i.e., it is linear with the supplied charge. Similarly, when applying 300 μA (Figure 5g), one can observe linear dependence but with a faster response time as the charge is entering the system faster (Figure 5h). However, the slope of the curve at 300 μA is approximately only seven times higher than that at 30 μA. This indicates side reactions, and therefore, the current is not entirely consumed to reduce Sn. However, the linear behavior of this sensor indicates that it can be used to quantify the amount of current passed through the sensor based on the color of the electrolyte. It is worth noting that the color shift may occur due to the pH change when the device is exposed to an alkaline environment. However, most of the foods are acidic or neutral,^48^ making false positives due to pH highly unlikely.

The proposed scheme is compatible with self-standing electrolytes, which would be beneficial for commercial devices. To do so, a hydrogel electrolyte was realized using agar (Figure S7a). The purple hydrogel changes its color around both electrodes when the current passes through it (Figure S7b). Blue coloring can be observed around the Sn wire due to the complex formation, while green coloring around Au can be ascribed to the formation of hydroxyl ions. Nevertheless, the green color is not stable and it quickly fades (Figure S7c,d) as anthocyanins exhibit low stability in basic solutions.^43,49^ However, the blue color persists even after a prolonged time in air, diffusing through the whole gel. The color change is irreversible, so the stored information is nonvolatile, as in a memory cell. The mechanism of the reaction was further investigated using different indicators such as phenolphthalein and quercetin, as discussed in the SI (Figure S8).^50^

### 2.3 Proof-of-Concept Device

Finally, to provide a complete proof of concept of a detector of defrosting events, the edible temperature-activated galvanic cell was coupled with the ionochromic cell. First, a device was assembled without adding the electrolyte (Figure S9). The edible galvanic cell was filled with an electrolyte first, and the electrolyte was rapidly frozen at −80 °C. Subsequently, the electrolyte chamber of the ionochromic cell was filled with the electrolyte containing red cabbage juice, and the devices were placed in the freezer (at around −21 °C). Following exposure to subzero temperatures overnight, the frozen sensors were placed at room temperature and the devices were allowed to operate for a certain amount of time. The electrolyte solutions of the color-changing galvanic sensor were collected after 5, 10, 20, and 30 min and characterized using the UV–vis spectra.

**Figure 6:**
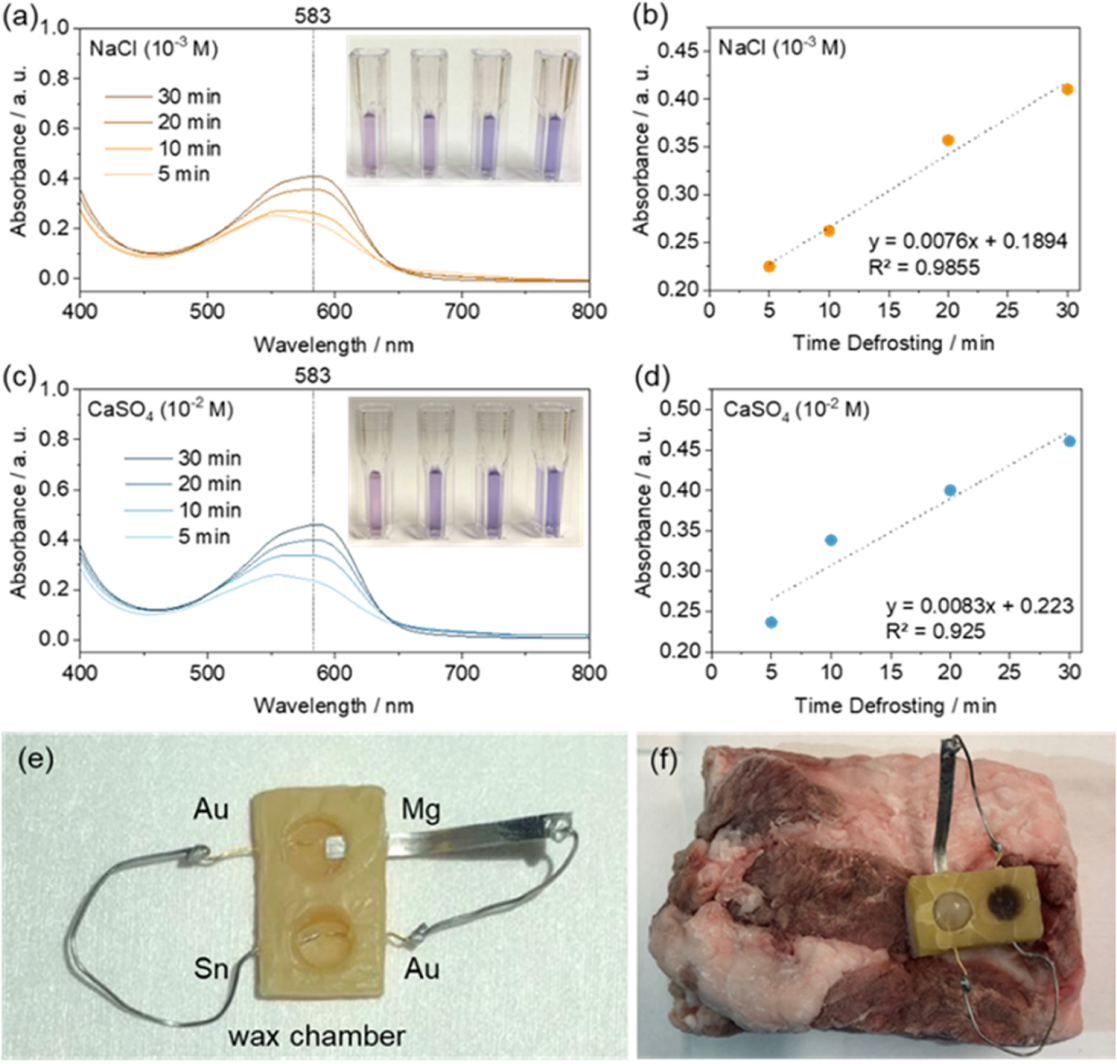
Edible defrosting sensor. (a, c) UV–vis spectra of the (a) 10^–3^ M NaCl and (c) 10^–2^ M CaSO_4_ electrolytes of the defrosting sensor after 5, 10, 20, and 30 min of operation. (b, d) Linear fit of the absorbance at 583 nm as a function of defrosting time. (e) Color-changing temperature sensor made of fully edible materials with the chamber made of wax. (f) Sensor on a piece of pork rind.

UV–vis spectra of the device containing 10^–3^ M NaCl exhibit an absorbance increase, with the maximum around 583 nm, as a function of time (Figure 6a). Comparing the absorption at the maximum, one can see a linear increase in the absorption with time (Figure 6b), indicating that the device follows the Beer–Lambert law. The latter observation indicates that such a device can be used not only to visually detect defrosting of the food products in the food chain but also to understand how long was the food exposed to temperatures above the melting point of ice, therefore realizing an edible time–temperature indicator. Furthermore, a similar test was performed with the device containing 10^–2^ M CaSO_4_ (Figure 6c,d). As established before, a 10^–2^ M CaSO_4_ electrolyte produces a higher current than 10^–3^ M NaCl. Therefore, the devices operated with CaSO_4_ exhibited a faster dynamics of the increase in optical absorbance with time, indicating that the response of the device is tunable by the concentration and nature of the electrolyte used.

We determined the stability of the device by keeping it in the freezer for 6 days and measuring the absorption of the ionochromic cell after 0 and 30 min of operation at room temperature (Figure S10). The results indicate a small leakage already while the device is in the freezer, forming a small amount of blue complex. Such a leakage is negligible compared to the amount of blue complex formed after 30 min of operation. However, the current leakage should be addressed in the future, as it could present problems when the sensor is used for longer times.

In these demonstrations, we used PDMS chambers for simplicity. As PDMS is a passive component, it can be effectively replaced with any water–insoluble material. As an example, we demonstrate that the sensor can be formed only with edible materials using a wax chamber instead of PDMS (Figure 6e). Such a system could be adapted for food monitoring (Figure 6f). The details of the edibility of each component of this device can be found in the SI.

## 3 Conclusions

In this work, we proposed a proof-of-concept of an edible self-powered defrosting sensor, which simply detects defrosting events or quantifies exposure to threshold temperatures. The device comprises an edible galvanic cell and an edible ionochromic cell.

The edible galvanic cell was made of magnesium and gold connected with an aqueous electrolyte containing an edible salt. The aqueous electrolyte freezing hinders ion transport and subsequently suppresses the current flow, which in turn is observable upon defreezing. We demonstrated the tunability of the current onset temperature using different salts and salt concentrations. We tuned the onset temperature from −50 to 0 °C. The mechanism of changing the offset temperature was clarified by correlating the electrochemical results with crystallinity. To our knowledge, the electrochemical conductivity of ice was not correlated with the crystallinity of ice before.

The current produced by the galvanic cell was successfully transduced into a visible signal with an ionochromic cell. The color-changing sensor is based on an electrochemical cell with tin and gold electrodes immersed in a solution of sodium chloride and red cabbage juice, an ionochrome. Such an electrochemical cell does not produce current spontaneously but undergoes electrochemical reactions once a current is applied. Upon applying the current, the device releases tin cations in the solution, which interact with anthocyanins from red cabbage, producing blue-colored complexes. Therefore, the galvanic cell coupled with the ionochromic cell forms a defrosting sensor device. Furthermore, we have shown that such a device also provides quantitative information about defrosting. Indeed, the concentration of tin complexes can be measured by UV–vis spectroscopy and correlated with the length of the exposure. Such an edible sensor could be introduced in food such as meat and fish, allowing the supply chain to monitor quantitatively, and the end consumers to monitor qualitatively, preservation quality. By tuning the current produced by the galvanic cell and the response produced by the electrochromic cell, it is possible to control the response time of the device. Therefore, it is possible to design devices with different activation times, as dictated by the product they are monitoring. Furthermore, we envision the change of the electrochromic cell with other galvanic indicators, which could emit a signal, making the reading of the device easier. Ease of readout could also be achieved with the integration of the galvanic cell in resonant structures, e.g., antennas.

This device is a proof of concept that such a sensor can be created using only edible materials. The miniaturization of such self-powered devices through smaller wax chambers, and printed thin films of metals and an optimized design will produce a fully edible sensor. When designing such a sensor, it is of crucial importance to isolate the chambers filled with electrolytes from the food. This sensor could pave the way for an inexpensive and safe technology to be largely exploited in the food and drug cold supply chains, reducing wastes and improving safety. We envision that the sensor could be especially useful for smart packaging, which can provide additional information about the product such as its storage condition history.

## 4 Materials and Methods

### 4.1 Materials

Magnesium ribbon (99.5%+), sodium chloride (99.0%+), agar (for microbiology), phenolphthalein indicator, tin(II) chloride (98%+), tin foil (99.9%+, 0.127 mm thick), and beeswax were purchased from Sigma-Aldrich. Calcium chloride dihydrate (99%+), calcium d-gluconate monohydrate (98%+), calcium sulfate dihydrate (98.0%+), and quercetin dihydrate (97%+) were acquired from Alfa Aesar. Sodium hydroxide concentrate (0.1 M in water) was purchased from Fluka. Tin wire (96.5% tin, with 3.0% silver, and 0.5% copper) was obtained from RS Components Ltd. Glycerol (99.0%+) was purchased from Merck. Gold wires (99.99%+) were acquired from 8853 SPA. Polydimethylsiloxane (PDMS), Sylgard 184, was purchased from Sigma-Aldrich. Apple, melon, grapes, and red cabbage were sourced from local supermarkets. The water used in all of the experiments was Milli-Q water.

### 4.2 X-ray Diffraction

X-ray diffraction (XRD) experiments were performed with a Bruker D8 ADVANCE diffractometer with Bragg–Brentano geometry, Cu Kα radiation (λ = 1.54056 Å), step increment of 0.02°, and 0.3 s of acquisition time. The sample was placed in an MTC-LOWTEMP chamber, where the temperature was controlled through an AlCr heater and a cold probe flushed with liquid nitrogen. A volume of 150 μL of the solution was added to the sample holder. The sample was first frozen down to a temperature of −80 °C at the rate of 1 °C min^–1^, and then the measurements were performed by heating the sample at the rate of 10 °C min^–1^, with thermalization for 5 min before starting the data acquisition at each selected temperature.

### 4.3 Ultraviolet–Visible Spectroscopy

Ultraviolet–visible (UV–vis) spectra were collected with a PerkinElmer LAMBDA 1050 UV–vis–NIR spectrophotometer in the range from 400 to 800 nm. The data were collected every 1 nm.

### 4.4 Electrochemical Experiments

All of the electrochemical experiments were performed on a MultiPalmSens4, a multichannel potentiostat/galvanostat/impedance analyzer in a two-electrode setup. Magnesium or tin were connected as working electrodes, and gold was connected as a counter/reference electrode. The experiments were performed in triplicate, and the standard error is indicated.

## Supporting Information Available

Images indicating the time elapsed since the electrolyte containing phenolphthalein was added in galvanic cells; open-circuit voltage of the galvanic cells; setup used for the investigation of the defrosting behavior of the electrolytes; chronoamperometry of cells with different calcium salts; X-ray diffractograms of water and 0.1 M aqueous solution of NaCl; color shift of red cabbage juice with addition of NaOH; tin–gold galvanic sensor with gel electrolyte; galvanic cells with phenolphthalein and quercetin indicators; edible galvanic cell–galvanic sensor couple; UV–vis absorption of the electrolyte from the ionochromic cell after 6 days of storage (PDF)
Operation of Mg–Au galvanic cell with 0.1 M NaCl as an electrolyte in open and closed circuit upon addition of the electrolyte (MP4)
