# Supplementary material for: Self-Powered Edible Defrosting Sensor
Source: ACS Sens. 2022 Oct 12;7(10):2995–3005. doi: 10.1021/acssensors.2c01280 (PMC9623588; doi:10.1021/acssensors.2c01280)
Supplement: Supplementary file 1 — se2c01280_si_001.pdf [file se2c01280_si_001.pdf]

Self-powered Edible Defrosting Sensor – Supplementary Information

Ivan K. Ilic,* Leonardo Lamanna, Daniele Cortecchia, Pietro Cataldi, Alessandro Luzio, and Mario Caironi*

Center for Nano Science and Technology@PoliMi, Istituto Italiano di Tecnologia, via Giovanni Pascoli 70/3 Milano 20133 Italy

ivan.ilic@iit.it, mario.caironi@iit.it

Edibility of the materials

Magnesium

Magnesium is an essential element in living systems. Magnesium salts can be bought as an over-the-counter dietary supplement. European Food Safety Authority (EFSA) defines an Adequate Intake of magnesium (ions) at 350 and 300 mg per day for men and women, respectively.[1] However, magnesium metal reacts with water in a relatively slow reaction producing hydrogen (rate of the reaction can be increased by microstructuring magnesium, lack of protective oxide layer, electrolytic nature of the solution, or reduced pH of water). Nevertheless, no side effects were reported for the oral administration of up to 200 mg of metallic magnesium.[2] Furthermore, an ingestible galvanic cell containing a small amount of metallic magnesium was approved for daily intake by the Food and Drug Administration (United States of America), and it received CE Mark designation (Europe).[3]

Gold

Although gold has no nutritional value, it is commonly used as decoration for food. While edible gold, 24 karat gold primarily in a leaf form, can be readily purchased in the European Union (EU) and it has designated code E 175 as a food additive, EFSA could not perform risk assessment due to the absence of toxicological data.[4]

Sodium chloride

Common table salt consists primarily of sodium chloride, the main source of electrolytes in European diets. EFSA set the reference values for sodium chloride at 5.1 g per day for adults.[5]

Calcium chloride

Calcium chloride is approved in the EU as food additive E 509. EFSA concluded that a reference value of 40 mg of chloride (equivalent to 63 mg of calcium chloride) per kilogram of body weight per day does not raise any safety concern.[6]

Calcium gluconate

Calcium gluconate is approved in the EU as food additive E 578.

Calcium sulfate

Calcium sulfate is approved in the EU as food additive E 516. EFSA does not provide any numerical acceptable daily intake for it as it raises no safety concern at the reported uses, far below 300 mg (sulfate-based) per kilogram of the body weight. At this level, sulfates are known to induce laxative effects.[7]

Tin

Widespread as food contact material, metallic tin can be found in the form of tin foil (replaced mainly by cheaper aluminum foil), tin cookware, and tin cans. Therefore, it is safe to assume that ingesting small

amounts of metallic tin can be deemed safe.[8] Furthermore, during electrochemical oxidation of tin in sodium chloride, we assume the formation of tin (II) chloride, commonly known as stannous chloride. EFSA approved it as a food additive, E 512, and the maximum permitted daily levels for stannous chloride are set at 25 mg of tin (40 mg of tin (II) chloride) per kg of the body weight.[9]

Agar
Agar is a substance obtained from algae widespread in food. In the EU, it is registered as food additive E 406. EFSA concluded there is no need for a numerical acceptable daily intake, as it showed no noticeable side effects after human consumption of 64 mg per kg of body weight for 12 weeks.[10]

Glycerol
Widespread in the food industry, glycerol is approved by the EU as E 422. EFSA concluded there is no need for a numerical acceptable daily intake of glycerol.[11]

Quercetin

Quercetin is a naturally occurring flavonoid found in many vegetables such as onions, kale, french beans, broccoli, lettuce, and tomatoes. It is among the highest consumed flavonoids, with the daily consumption estimated at 25-50 mg.[12] Furthermore, it can be bought as an over-the-counter food supplement.

Beeswax

Beeswax is a naturally occurring compounds, produced by bees. It is approved in EU as E 901 and it is a widely used food additive. The variety of toxicological studies set the no observed adverse effect levels no lower than 220 mg per kilogram of bodyweight.[13]

Preparation of materials and devices

Polydimethylsiloxane (PDMS) chambers

PDMS (*SYLGARD®184*) was mixed in a ratio of 10:1 with its curing agent, was poured in a glass Petri dish, degassed overnight, and baked at 100 °C for 2 h in the oven. Furthermore, the cavities were introduced in the formed PDMS chambers using a circular punch with a diameter of 1 cm. The chambers were cut in the desired shape, transferred to clean glass, and heated at 120° for 10 min to improve the adhesion to the glass. The small cavities were created on the sides of the chambers to accommodate the electrodes.

Juice of red cabbage

Red cabbage (100 g) was cut into small pieces using scissors and boiled with 200 mL of water. The hotplate was set at 250 °C, and the mixture was left to heat for approximately 30 min before cooling. The pieces of red cabbage were filtrated through a fiberless paper, while the juice was collected and diluted to reach a total volume of 200 mL.

Hydrogel electrolytes containing the red cabbage juice

The hydrogels were prepared by dissolving sodium chloride (116 mg) in the juice of red cabbage (15 mL). After stirring for 1 h at 80 °C, glycerol was added to the solution, and it was further mixed at the same temperature for 10 min. Afterward, the obtained solution was cast in a glass petri dish, left to cool to RT, and then at 5 °C for at least 1 h before being used.

Hydrogel electrolytes containing the indicators

The hydrogels were prepared by dissolving sodium chloride (116 mg) in water (15 mL). The indicator was then added, either phenolphthalein (10 µL of the 10 mg/mL solution in ethanol) or quercetin dihydrate (1 mg). After stirring for 1 h at 80 °C, glycerol was added to the solution, and it was further mixed at the same temperature for 10 min. Afterward, the obtained solution was cast in a glass petri dish, left to cool to RT, and then at 5 °C for at least 1 h before being used.

Electrolyte containing red cabbage juice

Sodium chloride (23 mg) was dissolved in red cabbage juice (3 mL) and glycerol (1 mL).

Assembly of the sensor

The complete sensor was assembled inside the PDMS chamber containing two electrolyte cavities. Magnesium ribbon and gold wire were put in the first cavity and gold and tin wire in the second cavity. Gold wire (from the Mg-Au cell) was soldered with the tin wire. Magnesium ribbon and gold wire (from Sn-Au cell) were connected with the crocodile clip. The cavity of the Mg-Au cell was firstly filled with the electrolyte (0.4 mL), followed by freezing the cell at -80 °C for 20 min. Afterward, electrolyte-containing red cabbage juice (0.4 mL) was added to fill the Sn-Au cell, and the device was put in a kitchen-grade freezer (around -21 °C) overnight.

Assembly of vax-based temperature sensor

Beeswax was melted at 80 °C. Afterward, it was cast into a mold including two PDMS cylinders (1 cm in diameter and high) as a template. Upon cooling the wax and removing the PDMS, the sensor was prepared for the tests as described in *Assembly of the sensor*.

Details of the experiments

UV/Vis with NaOH

The juice of red cabbage (0.2 mL) was diluted with water (0.8 mL). Furthermore, different amounts of the aqueous solution of sodium hydroxide (0.1 M) were added: 0, 2, 4, 6, 10, 15, or 20 µL. The change in the absolute volume was considered negligible.

UV/Vis with tin chloride

The juice of red cabbage (0.2 mL) was diluted with water (0.8 mL). Furthermore, different amounts of solution of tin (II) chloride (0.02 M) in ethanol were added: 0, 2, 4, 6, 10, or 15 µL, along with pure ethanol: 20, 18, 16, 14, 10, or 5 µL.

Mg-Au cell

Magnesium stripe and gold wire were inserted on the opposite sides of the PDMS chamber so that each metal enters around 2 mm in the chamber cavity. The cavities were filled with the appropriate electrolyte (0.4 mL) right before the beginning of the electrochemical experiment. The chronoamperometry and open-circuit voltage were recorded for 30 minutes. Electrochemical Impedance Spectroscopy (EIS) was measured from 1 mHz to 1 MHz.

Defrosting of the Mg-Au cell

The Mg-Au cell was prepared as described previously, but it was put in the freezer at -80 °C immediately after the addition of the electrolyte and kept at the temperature overnight to ensure the whole system was

cooled down to -80 °C. Upon taking the samples out of the fridge, the chronoamperometry experiments were performed on the cells. The time in these experiments represents the time elapsed since the cells were taken out of the freezer. Therefore, the first 30 seconds are not shown in any of the experiments as it is the time it takes to take the cells from the freezer and connect them to the measuring device.

Fruit as electrolytes

Grapes, apples, and melon were used as electrolytes. Magnesium ribbon and gold wire were inserted inside the fruit. Afterward, the cells were frozen and tested as explained previously in *Defrosting of the Mg-Au cell*.

Current measured at different temperatures

The galvanic cells were made by inserting the magnesium ribbon and gold electrode (connected with the protected wire) in a vial (Figure S3a). After adding the electrolyte (3 mL), the system was sealed using parafilm, and 4 cells were immersed in ethanol equipped with a stirring bar (Figure S3b). The system was cooled by inserting it in the freezer at -80 °C. Upon cooling, the system was connected to the potentiostat and the thermocouple was immersed in the ethanol to measure the temperature. The ethanol was stirred, and the temperature was allowed to rise. At chosen temperatures, the chronoamperometry experiments were performed for 15 s. The temperature indicated is at the beginning of the measurement, while the current indicates the last data point as obtained in the chronoamperometry experiment. Once the temperature of the mixture reached the new value, the same chronoamperometry experiment was repeated.

UV/Vis of the electrolyte containing red cabbage juice after the applied current

The cell containing tin and gold wires was created. The electrolyte containing red cabbage juice (0.4 mL) was added, and the current of 30 μA or 300 μA was applied for a defined period using the chronopotentiometry technique. The electrolyte was homogenized upon finishing the experiment, and a 0.2 mL aliquot was added to the water (0.8 mL).

Applying the current at the hydrogel electrolytes containing the indicators

The hydrogel electrolytes containing the indicators were pierced with a tin wire electrode and gold wire electrode. The current of 1 mA was applied for 60 s using the chronopotentiometry technique.

UV/Vis of the temperature sensor

The sensor was assembled as described in *Assembly of the sensor*. After the sensors were taken out of the freezer they were left at room temperature to operate for a certain amount of time before the 0.2 mL aliquot was taken and diluted with water (0.8 mL).

Stability of the temperature sensor

The sensor was assembled as described in the *UV/Vis of the temperature sensor* and the UV/Vis absorption of the electrolyte was performed for the sensor, previously stored in the freezer at -20 °C for 6 days, operated for 0 and 30 min at room temperature. Results are compared to the absorption of the pristine electrolyte.

Supplementary figures

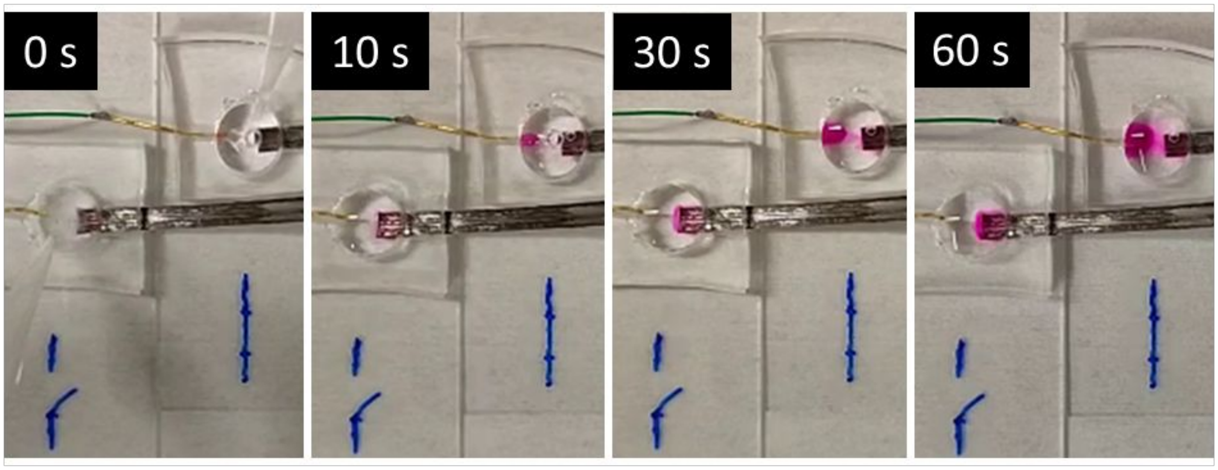

Figure S1. Galvanic cells consisting of the gold wire and the magnesium stripe immersed in the 0.1 M aqueous solution of sodium chloride containing phenolphthalein. The left cell has the open circuit while the right frame has the open circuit. The time indicates time elapsed since the electrolyte was added in the cells.

The reactions occurring in the galvanic cell by filling the cell with a 0.1 M aqueous solution of sodium chloride with phenolphthalein (Figure S1). Phenolphthalein is colorless at neutral pH but changes the color over the alkaline pH range 8.2 – 10. Two galvanic cells, one with the closed circuit and the other with the open circuit, were compared during 1 minute. In the closed-circuit galvanic cell, the purple color appears primarily in the region around the gold wire indicating formation of hydroxyl ions, confirming water reduction occurs on the surface of the gold wire. However, a small amount of pink coloring also occurs around the magnesium stripe, indicating a reaction between the magnesium and the water. In the open-circuit galvanic cell, there is no coloring around the gold wire, indicating a lack of reaction. However, intensive pink color can be observed around the magnesium stripe revealing the reaction of the magnesium and water. Intriguingly, pink coloring around magnesium is much more evident in the open-circuit cell, and therefore we conclude that the reaction of magnesium and water occurs slower during the electrochemical discharge of the cell.

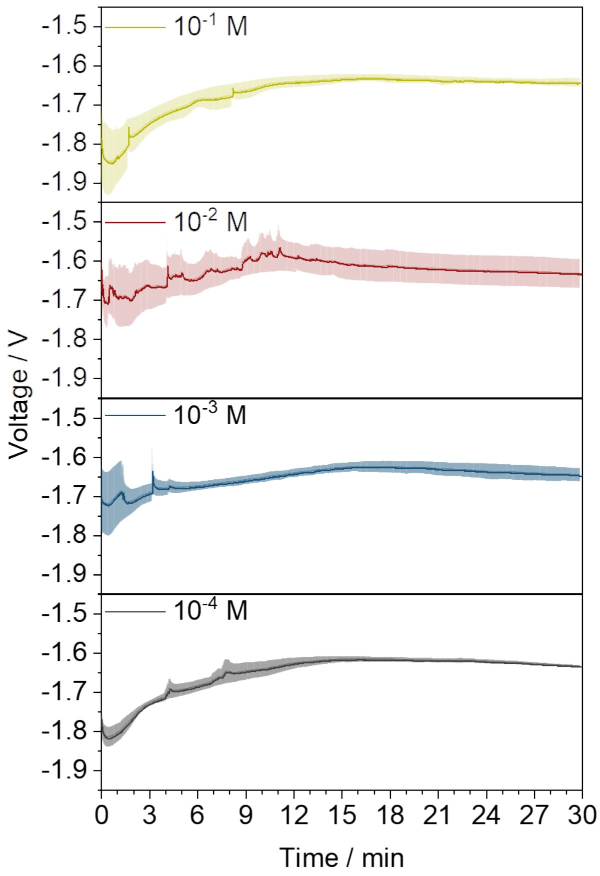

Figure S2. Open circuit voltage of the galvanic cells consisting of the gold wire and the magnesium stripe immersed in the aqueous solutions of sodium chloride with varying concentrations.

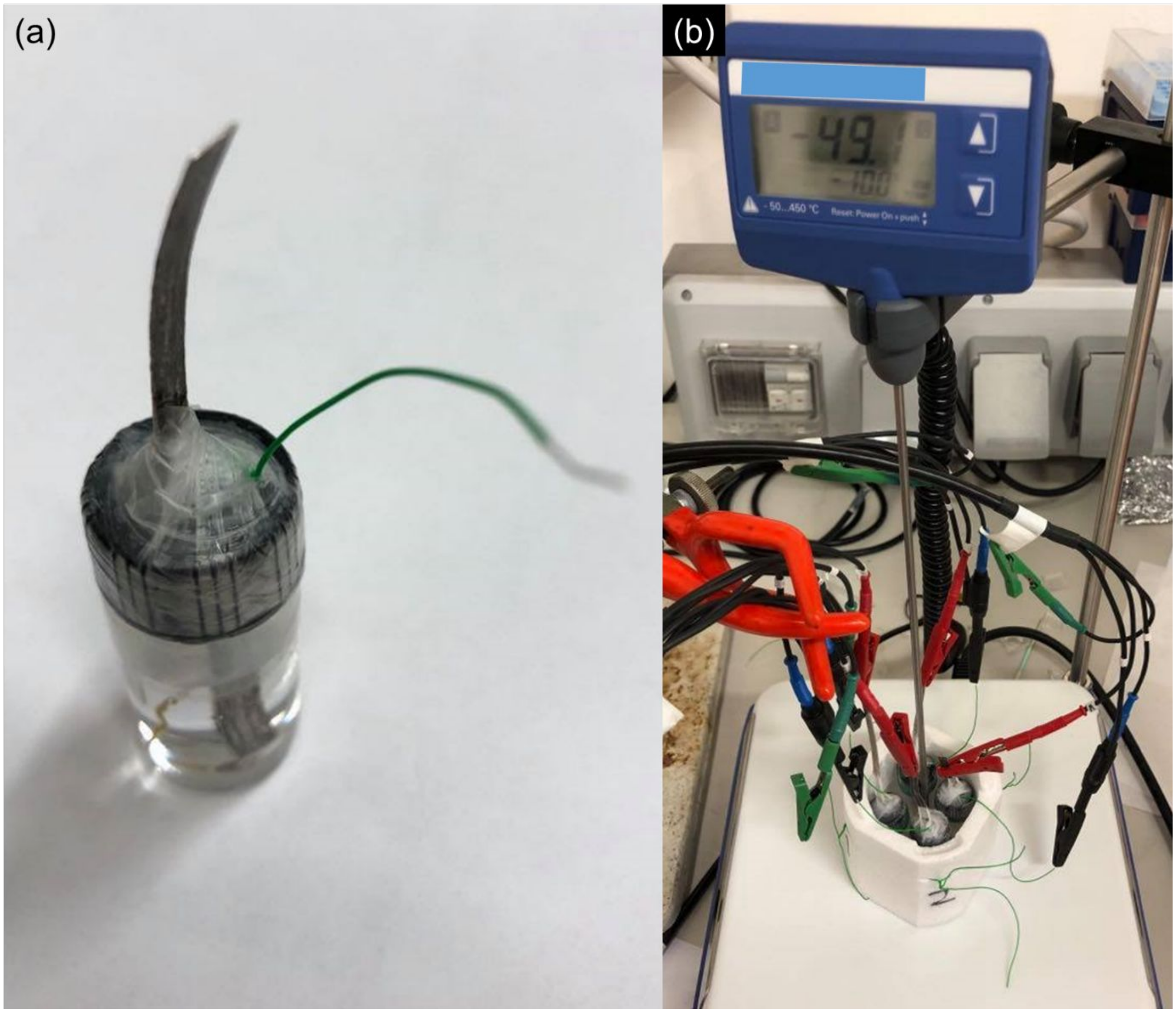

Figure S3. Setup used for the investigation of the defrosting behavior of the electrolytes. (a) Galvanic cell in the vial used in the experiment. (b) Styrofoam cup containing 4 galvanic cells, filled with ethanol to buffer the temperature change, and equipped with thermocouple to monitor the temperature.

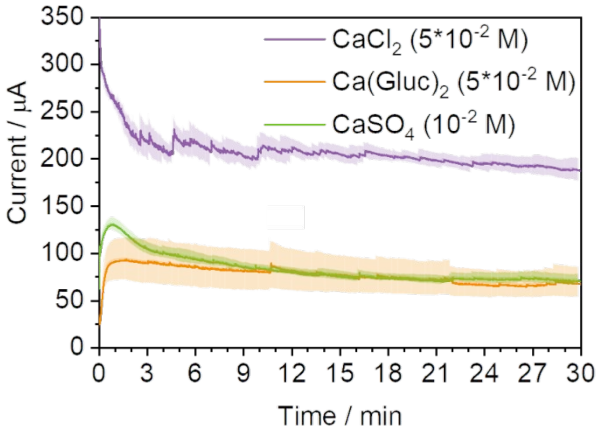

Figure S4. Chronoamperometry of the closed-circuit galvanic cells filled with different calcium salts as electrolytes.

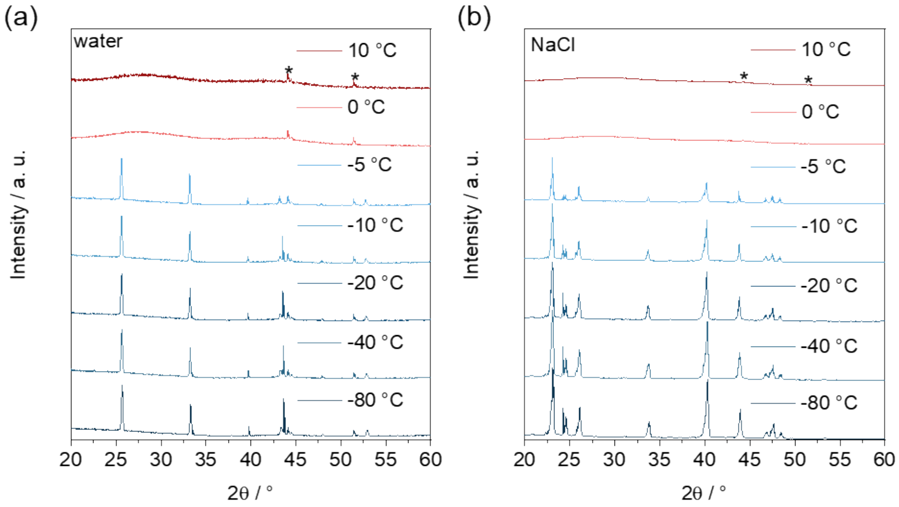

Figure S5. X-ray diffractograms of the water (a) and 0.1 M aqueous solution of sodium chloride (b) at different temperatures. The stars around reflections at 44° and 51° are result from (111) and (200) reflections of stainless steel sample holder.

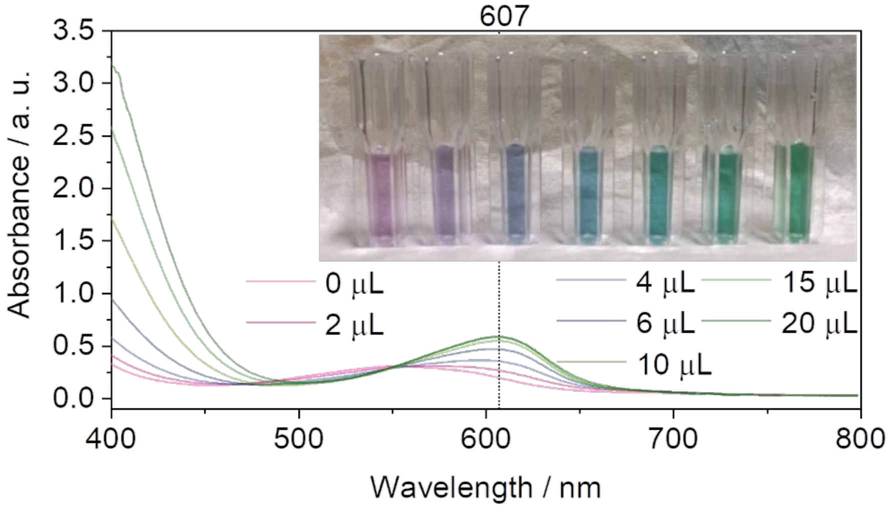

Figure S6 Color shift of red cabbage juice upon addition small portion of 0.1 M aqueous solution of sodium hydroxide as measured by ultraviolet–visible (UV-Vis) spectroscopy and photographed (insert).

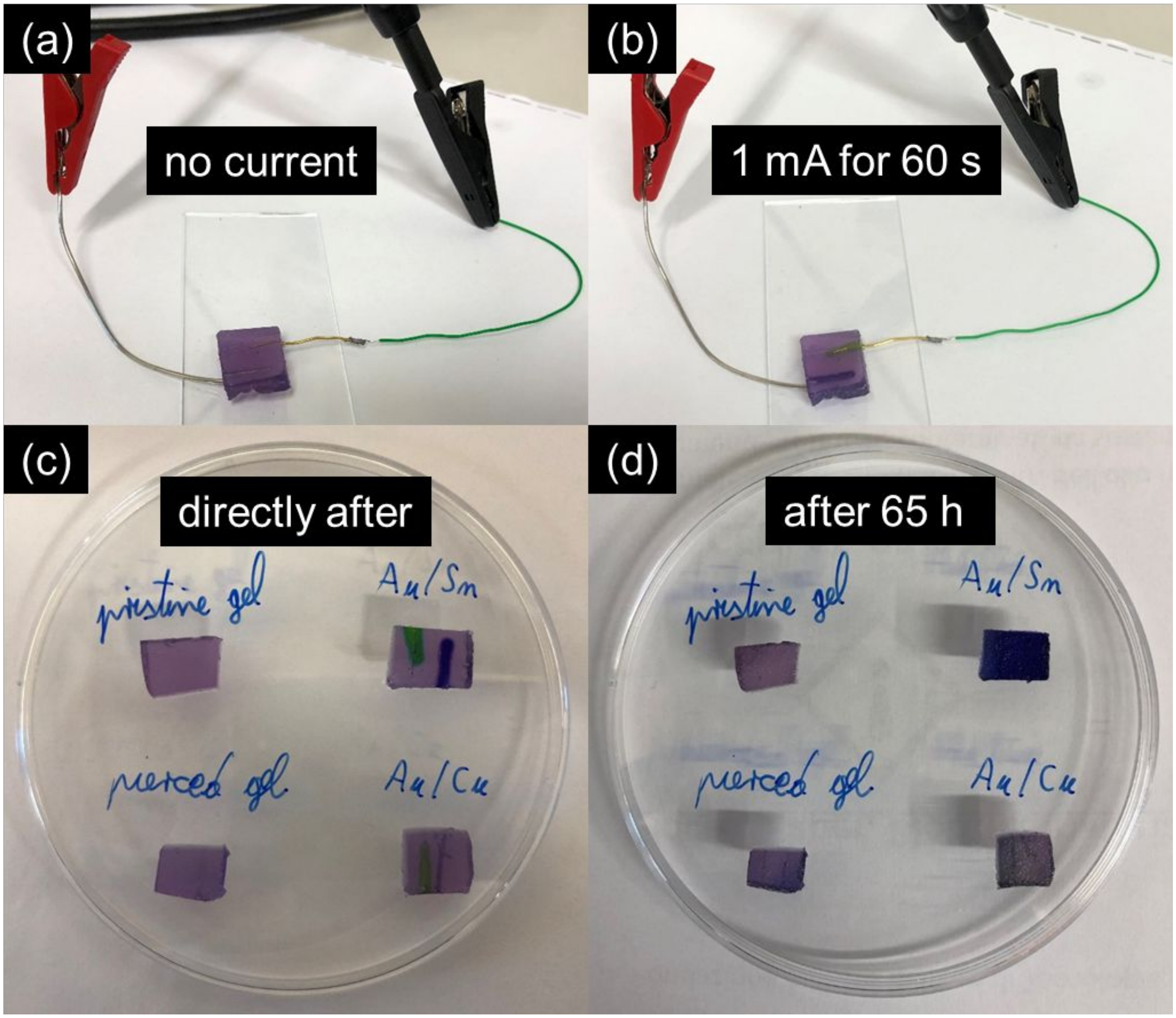

Figure S7. Tin-gold galvanic sensor with 3 w% agar hydrogel containing 0.1 M sodium chloride, red cabbage juice (75 vol%), and glycerol (25 vol%) as electrolyte. a) Galvanic sensor before the initiation of current. b) Galvanic sensor after passing 1 mA for 60 s. c) Sensor from b) compared to pristine gel, pierced gel (with tin and gold) and sensor with copper-gold galvanic sensor. d) The sensor and other gels for comparison after 65 h in air at room temperature.

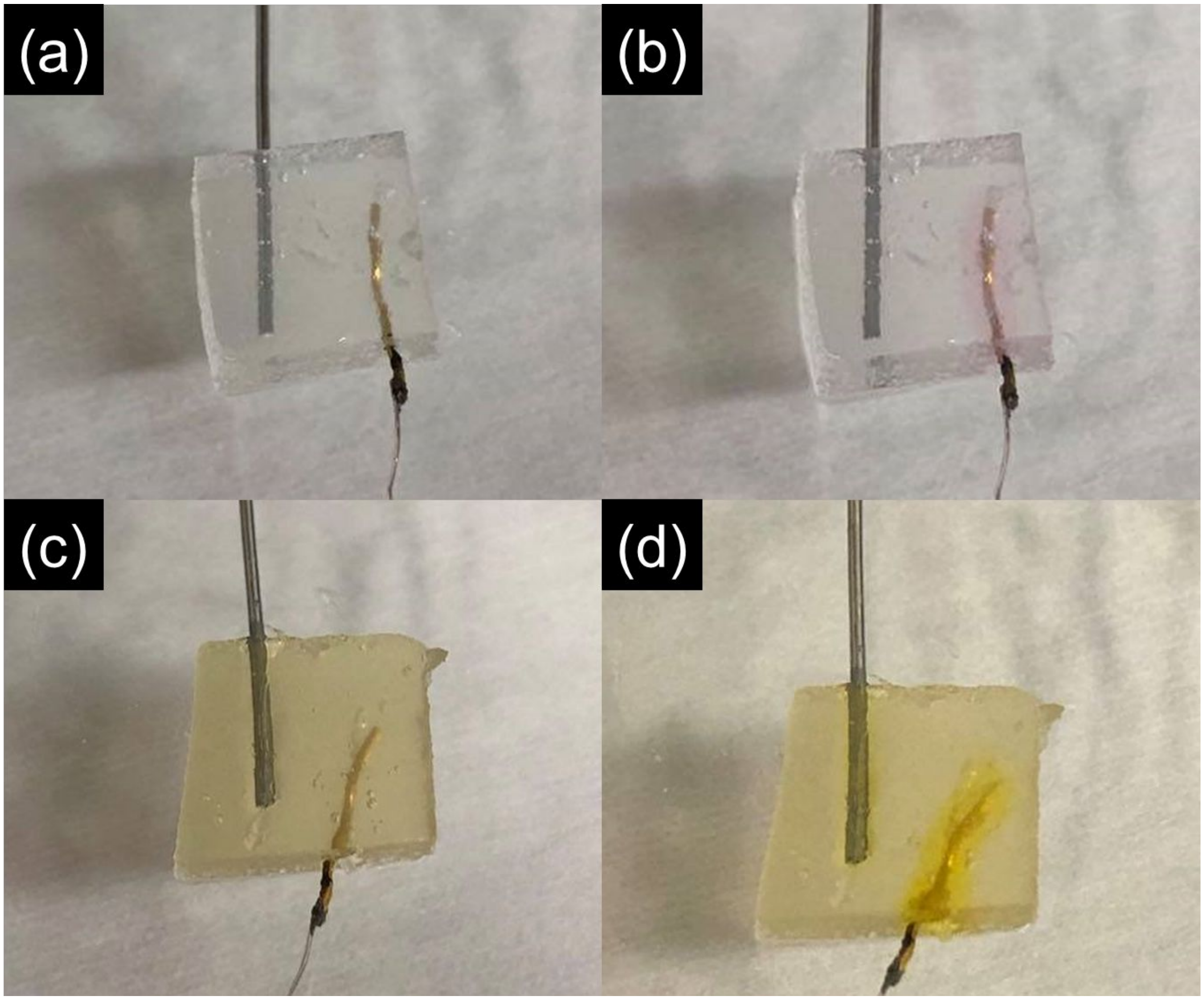

Figure S8. Galvanic cells with hydrogels containing phenolphthalein indicator (a,b) and quercetin indicator (c,d) before (a,c) and after (b,d) application of 1 mA for 60 seconds.

A galvanic sensor made from copper-gold electrodes did not exhibit blue coloring around the copper. This can be ascribed to a much lower sensitivity of anthocyanins to copper, as compared to other metals such as tin.[14] Furthermore, hydrogels containing phenolphthalein and quercetin indicators were utilized to prove the importance of anthocyanin complexes with tin (Figure S10). Transparent hydrogels containing phenolphthalein undergo coloration to purple around the gold electrode when the current is applied, while the tin electrode remains colorless (Figure S10a,b). This indicates basic region around gold, in agreement with the proposed reaction, while tin forms no complex with phenolphthalein. However, pale-yellow gels of quercetin change the color to deep yellow both around the gold electrode, and, to a smaller extend, around the tin electrode (Figure S9c,d). Quercetin is an flavonoid that undergoes color change from pale yellow to deep yellow in quinoidal form, due to the bathochromic shift with the rising pH.[15] On the gold electrode, the change is observed due to the increase in pH while the tin electrode releases tin ions which form complexes with tin. This is in agreement that tin-anthocyanin complex changes the color of red cabbage to blue.

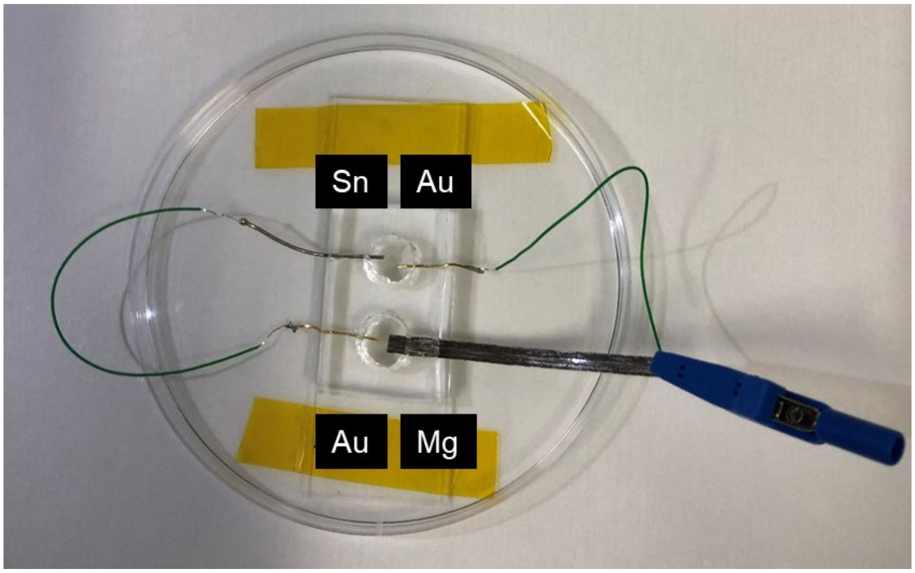

Figure S9. Edible galvanic cell – galvanic sensor couple used for the experiments prior to the filling of the chambers with the electrolytes.

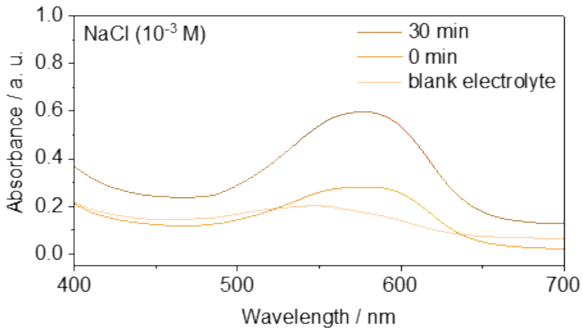

Figure S10. UV/Vis absorption of the electrolyte from the ionochromic cell. Represented are: absorption of the electrolyte as filled in the cell (blank electrolyte), absorption of the electrolyte after 6 days storage of the sensor in a freezer (0 min); absorption of the electrolyte after 6 days storage of the sensor in device freezer and sensor operation at room temperature for 30 min (30 min).

Video S1. Operation of Mg-Au galvanic cell with 0.1 M NaCl as an electrolyte in open and closed circuit upon addition of the electrolyte.

References

(1) Agostoni, C.; Berni Canani, R.; Fairweather-Tait, S.; Heinonen, M.; Korhonen, H.; La Vieille, S.; Marchelli, R.; Martin, A.; Naska, A.; Neuhäuser-Berthold, M.; Nowicka, G.; Sanz, Y.; Siani, A.; Sjödin, A.; Stern, M.; Strain, S. (J. J. .; Tetens, I.; Tomé, D.; Turck, D.; Verhagen, H. Scientific Opinion on Dietary Reference Values for Magnesium. *EFSA J.* **2015**, *13* (7), 4186. https://doi.org/10.2903/j.efsa.2015.4186.

(2) Sack, D. A.; Stephensen, C. B. Liberation of Hydrogen from Gastric Acid Following Administration of Oral Magnesium. *Dig. Dis. Sci.* **1985**, *30* (12), 1127–1133. https://doi.org/10.1007/BF01314045.

(3) Hafezi, H.; Robertson, T. L.; Moon, G. D.; Au-Yeung, K. Y.; Zdeblick, M. J.; Savage, G. M. An Ingestible Sensor for Measuring Medication Adherence. *IEEE Trans. Biomed. Eng.* **2015**, *62* (1),

99–109. https://doi.org/10.1109/TBME.2014.2341272.

(4) Aguilar, F.; Crebelli, R.; Di Domenico, A.; Dusemund, B.; Frutos, M. J.; Galtier, P.; Gott, D.; Gundert-Remy, U.; Lambré, C.; Leblanc, J.-C.; Lindtner, O.; Moldeus, P.; Mortensen, A.; Mosesso, P.; Oskarsson, A.; Parent-Massin, D.; Stankovic, I.; Waalkens- Berendsen, I.; Woutersen, R. A.; Wright, M.; Younes, M. Scientific Opinion on the Re-evaluation of Gold (E 175) as a Food Additive. *EFSA J.* **2016**, *14* (1), 4362. https://doi.org/10.2903/j.efsa.2016.4362.

(5) Turck, D.; Castenmiller, J.; de Henauw, S.; Hirsch-Ernst, K. I.; Kearney, J.; Knutsen, H. K.; Maciuk, A.; Mangelsdorf, I.; McArdle, H. J.; Pelaez, C.; Pentieva, K.; Siani, A.; Thies, F.; Tsabouri, S.; Vinceti, M.; Aggett, P.; Fairweather-Tait, S.; Martin, A.; Przyrembel, H.; de Sesmaisons-Lecarré, A.; Naska, A. Dietary Reference Values for Chloride. *EFSA J.* **2019**, *17* (9), 5779. https://doi.org/10.2903/j.efsa.2019.5779.

(6) Younes, M.; Aquilina, G.; Castle, L.; Engel, K. H.; Fowler, P.; Fürst, P.; Gürtler, R.; Gundert-Remy, U.; Husøy, T.; Mennes, W.; Moldeus, P.; Oskarsson, A.; Shah, R.; Waalkens-Berendsen, I.; Wölfle, D.; Boon, P.; Crebelli, R.; Di Domenico, A.; Filipič, M.; Mortensen, A.; Van Loveren, H.; Woutersen, R.; Giarola, A.; Lodi, F.; Riolo, F.; Frutos Fernandez, M. J. Re-Evaluation of Hydrochloric Acid (E 507), Potassium Chloride (E 508), Calcium Chloride (E 509) and Magnesium Chloride (E 511) as Food Additives. *EFSA J.* **2019**, *17* (7), 5751. https://doi.org/10.2903/j.efsa.2019.5751.

(7) Younes, M.; Aquilina, G.; Castle, L.; Engel, K. H.; Fowler, P.; Fürst, P.; Gürtler, R.; Gundert-Remy, U.; Husøy, T.; Mennes, W.; Moldeus, P.; Oskarsson, A.; Shah, R.; Waalkens-Berendsen, I.; Wölfle, D.; Boon, P.; Crebelli, R.; Di Domenico, A.; Filipič, M.; Mortensen, A.; Van Loveren, H.; Woutersen, R.; Giarola, A.; Lodi, F.; Riolo, F.; Frutos Fernandez, M. J. Re-Evaluation of Sulphuric Acid and Its Sodium, Potassium, Calcium and Ammonium Salts (E 513, 514 (i), 514 (Ii), 515 (i), 515 (Ii), 516 and 517) as Food Additive. *EFSA J.* **2019**, *17* (10), 5868. https://doi.org/10.2903/j.efsa.2019.5868.

(8) Blunden, S.; Wallace, T. Tin in Canned Food: A Review and Understanding of Occurrence and Effect. *Food Chem. Toxicol.* **2003**, *41* (12), 1651–1662. https://doi.org/10.1016/S0278-6915(03)00217-5.

(9) Younes, M.; Aggett, P.; Aguilar, F.; Crebelli, R.; Dusemund, B.; Filipič, M.; Frutos, M. J.; Galtier, P.; Gott, D.; Gundert-Remy, U.; Kuhnle, G. G.; Lambré, C.; Leblanc, J. C.; Lillegaard, I. T.; Moldeus, P.; Mortensen, A.; Oskarsson, A.; Stankovic, I.; Waalkens-Berendsen, I.; Wright, M.; Di Domenico, A.; Van Loveren, H.; Giarola, A.; Horvath, Z.; Lodi, F.; Riolo, F.; Woutersen, R. A. Re-Evaluation of Stannous Chloride (E 512) as Food Additive. *EFSA J.* **2018**, *16* (6), 5295. https://doi.org/10.2903/j.efsa.2018.5295.

(10) Mortensen, A.; Aguilar, F.; Crebelli, R.; Di Domenico, A.; Frutos, M. J.; Galtier, P.; Gott, D.; Gundert-Remy, U.; Lambré, C.; Leblanc, J.; Lindtner, O.; Moldeus, P.; Mosesso, P.; Oskarsson, A.; Parent-Massin, D.; Stankovic, I.; Waalkens-Berendsen, I.; Woutersen, R. A.; Wright, M.; Younes, M.; Brimer, L.; Peters, P.; Wiesner, J.; Christodoulidou, A.; Lodi, F.; Tard, A.; Dusemund, B. Re-evaluation of Agar (E 406) as a Food Additive. *EFSA J.* **2016**, *14* (12), 4645. https://doi.org/10.2903/j.efsa.2016.4645.

(11) Mortensen, A.; Aguilar, F.; Crebelli, R.; Di Domenico, A.; Dusemund, B.; Frutos, M. J.; Galtier, P.; Gott, D.; Gundert-Remy, U.; Leblanc, J.; Lindtner, O.; Moldeus, P.; Mosesso, P.; Parent-Massin, D.; Oskarsson, A.; Stankovic, I.; Waalkens-Berendsen, I.; Woutersen, R. A.; Wright, M.; Younes, M.; Boon, P.; Chrysafidis, D.; Gürtler, R.; Tobback, P.; Rincon, A. M.; Tard, A.; Lambré, C. Re-evaluation of Glycerol (E 422) as a Food Additive. *EFSA J.* **2017**, *15* (3), 4720. https://doi.org/10.2903/j.efsa.2017.4720.

(12) Formica, J. V.; Regelson, W. Review of the Biology of Quercetin and Related Bioflavonoids. *Food Chem. Toxicol.* **1995**, *33* (12), 1061–1080. https://doi.org/10.1016/0278-6915(95)00077-1.

(13) Aguilar, F.; Autrup, H.; Barlow, S.; Castle, L.; Crebelli, R.; Engel, K.; Gontard, N.; Gott, D.; Grilli, S.; Gürtler, R.; Larsen, C.; Leclercq, C.; Leblanc, J.; Malcata, F. X.; Mennes, W.; Milana, M. R.; Pratt, I.; Rietjens, I.; Tobback, P.; Toldrá, F. Scientific Opinion of the Panel on Food Additives , Flavourings , Processing Aids and Materials in Contact with Food Adopted on 7 March 2008. *EFSA J.* **2007**, *615* (80), 1–28.

(14) Starr, M. S.; Francis, F. EFFECT OF METALLIC IONS ON COLOR AND PIGMENT CONTENT OF CRANBERRY JUICE COCKTAIL. *J. Food Sci.* **1973**, *38* (7973), 1043–1046.

(15) Jurasekova, Z.; Domingo, C.; Garcia-Ramos, J. V.; Sanchez-Cortes, S. Effect of PH on the Chemical Modification of Quercetin and Structurally Related Flavonoids Characterized by Optical (UV-Visible and Raman) Spectroscopy. *Phys. Chem. Chem. Phys.* **2014**, *16* (25), 12802–12811. https://doi.org/10.1039/c4cp00864b.
